# The Role of PTEN in Chronic Growth Hormone-Induced Hepatic Insulin Resistance

**DOI:** 10.1371/journal.pone.0068105

**Published:** 2013-06-28

**Authors:** Yuan Gao, Peizhu Su, Chuqiong Wang, Kongqin Zhu, Xiaolan Chen, Side Liu, Jiman He

**Affiliations:** 1 Department of Gastroenterology, Guangdong Provincial Key Laboratory of Gastroenterology, Nanfang Hospital, Southern Medical University, Guangzhou, China; 2 Liver Research Center, Brown University, Providence, Rhode Island, United States of America; Wayne State University, United States of America

## Abstract

Chronic growth hormone (GH) therapy has been shown to cause insulin resistance, but the mechanism remains unknown. PTEN, a tumor suppressor gene, is a major negative regulator of insulin signaling. In this study, we explored the effect of chronic GH on insulin signaling in the context of PTEN function. Balb/c healthy mice were given recombinant human or bovine GH intraperitoneally for 3 weeks. We found that phosphorylation of Akt was significantly decreased in chronic GH group and the expression of PTEN was significantly increased. We further examined this effect in the streptozotocin-induced Type I diabetic mouse model, in which endogenous insulin secretion was disrupted. Insulin/PI3K/Akt signaling was impaired. However, different from the observation in healthy mice, the expression of PTEN did not increase. Similarly, PTEN expression did not significantly increase in chronic GH-treated mice with hypoinsulinemia induced by prolonged fasting. We conducted in-vitro experiments in HepG2 cells to validate our in-vivo findings. Long-term exposure to GH caused similar resistance of insulin/PI3K/Akt signaling in HepG2 cells; and over-expression of PTEN enhanced the impairment of insulin signaling. On the other hand, disabling the PTEN gene by transfecting the mutant PTEN construct C124S or siPTEN, disrupted the chronic GH induced insulin resistance. Our data demonstrate that PTEN plays an important role in chronic-GH-induced insulin resistance. These findings may have implication in other pathological insulin resistance.

## Introduction

Growth hormone (GH) therapy has been widely used in patients with growth deficiencies. However, excess GH has been demonstrated to be associated with the development of insulin resistance [1]–[3]. Transgenic mice over-expressing GH are suffered from hyperinsulinemia, and insulin resistance [4]. Chronic GH treatment has increased the incidence of type 2 diabetes by six folds in children [5]. The development of insulin resistance and diabetes, under the condition of chronic excessive GH, is at least partially attributable to the interference of GH with insulin signaling [6]. However, the detailed mechanisms have not been fully elucidated.

Insulin resistance is a condition in which normal amounts of insulin fail to elicit a typical insulin response from liver, fat, and muscle cells. In liver, insulin resistance leads to impaired glycogen synthesis and failure to suppress glucose production. These processes are regulated by insulin. It binds to the insulin receptor located on the outer surface of the plasma membrane via IRS-1 so as to activate phosphoinositide 3-kinase (PI3K) and Akt. Upon activation, Akt is phosphorylated and glycogen synthase kinase 3 (GSK-3), an inhibitory kinase, is inactivated, through which glycogen synthesis is regulated [7].

Several components of the insulin/PI3K pathway have been demonstrated to be involved in the development of insulin resistance upon chronic exposure to GH in several models. IRS-1 and PI3K protein levels have been reported to be decreased in the liver of GH-treated rats [8]; the extent of insulin-stimulated phosphorylation of insulin receptor, IRS-1/2, and PI3K have been demonstrated to fall in the liver and skeletal muscle of GH-treated rats and GH-transgenic mice [9], [10]. However, it remains unknown whether PTEN, the major negative regulator of the insulin/PI3K pathway, is involved in chronic GH therapy induced insulin resistance.

PTEN (+/−) mice exhibit similar increase in insulin sensitivity [11], and PTEN polymorphisms have been identified in type 2 diabetic patients [12]. We have recently demonstrated that acute ethanol treatment can increase the interaction of PTEN with p85α regulatory subunit of PI3K, resulting in the impairment of insulin signaling [13], [14]. In this study, we explored the effect of chronic GH on insulin signaling in the context of PTEN function.

## Materials and Methods

### 1. Antibodies and Reagents

p-Akt (Ser 473) (sc-7985), Akt (sc-8312), PI3K p85α (N-18) (sc-31969), PTEN (N-19) (sc-6818), p-PI 3-kinase p85α (Tyr 508) (sc-12929) antibodies were purchased from Santa Cruz Biotechnology, Inc. (Santa Cruz, CA). Phosphotyrosine (06–427) antibody was obtained from EMD Millipore Corporation (Billerica, MA). PI3K p85 (4257), phospho-p85 (tyr 458) antibodies were purchased from Cell Signaling, Inc. (Beverly, MA). PTEN (ALX-804-254-C100) antibody was obtained from ENZO Life Sciences, Inc. GAPDH (TA-08), Actin (TA-09) antibodies were purchased from Beijing Zhong Shan -Golden Bridge Biological Technology CO. Recombinant human GH was obtained from Shanghai United Cell Biotechnology Co. Bovine GH (30C-CP2042) was obtained from Fitzgerald. Streptozotocin (S-0130) was obtained from sigma. Recombinant human insulin was purchased from Lilly France. Western blots were developed with the use of a reagent inducing chemiluminescence (the ECL reagent; Beyotime Institute of Biotechnology, Nanjing, China or Millipore Corporation, Billerica, MA, USA). Protein A/G beads were purchased from Santa Cruz Biotechnology Inc. The plasmids HA-PTEN and HA-PTEN-C124S were kindly provided by Dr. William R. Sellers [15]. Mouse Insulin ELISA Kit (E05071m) was obtained from Cosmo Bio USA, Inc. Lipofectamine 2000 (11668-019) was purchased from Life Technologies Corporation. The small interfering RNAs targeting PTEN (siPTEN) were developed from Qiagen-Xeragon (Germantown, MD), the sense strand was 5′-GACUUGAAGGCGUAUACAGtt-3′, and the antisense strand was 5′-CUGUAUACGCCUUCAAGUCtt-3′.

### 2. Animal Care and GH Administration

Balb/c healthy male mice aging 6 to 8 weeks were housed in a standard animal facility, under conditions of controlled temperature and humidity, under a 12 h–12 h light-dark cycle, with free access to chow and water. Our research protocol was approved by the Committee on the Ethics of Animal Experiments of Southern Medical University. All animals were cared for in a humane manner, as outlined in the University Guide for Care and Use of Laboratory Animals. Mice were chronically treated with recombinant human growth hormone (hGH) or bovine growth hormone (bGH) intraperitoneally (1 µg/g body weight) daily (controls received saline) for 3 weeks, and the animals were further injected with insulin (2 IU/kg) or saline at the same volume 10 minutes before sacrifice.

C57BL/6 male mice aging 6 to 8 weeks were randomly divided into STZ and non-STZ groups. The STZ Na-citrate buffer (pH4.5) was prepared immediately before injection. STZ group received multiple intraperitoneal injections (for 3 consecutive days) of STZ (120 mg/kg) after an 8 hour fast, while non-STZ group received 10 mM Na-citrate buffer (pH 4,5) intraperitoneally. Following the 3-day administration of STZ, morning blood glucose was measured by tail-vein sampling using OneTouch® UltraVue™ glucometer. The diabetic mice were randomly divided into control group and chronic GH group, receiving 3-week daily administration of saline or hGH (1 µg/g body weight). Finally the mice were injected insulin (2I U/kg) or saline 10 minutes before sacrifice.

Given that STZ could damage pancreatic function, we further used a more physiological animal model. Balb/c male mice aging 6 to 8 weeks received 3-week daily administration of saline or hGH (1 µg/g body weight) and in the last 3 days, were exposed to 3-day fasting. Finally the mice were challenged with 10-min saline before sacrifice.

All surgery was performed using intraperitoneal sodium pentobarbital anesthesia.

### 3. Immunoprecipitation and Immunoblotting

For immunoprecipitation studies, liver tissue was homogenized in PBS containing protease and phosphatase inhibitors. The lysate was centrifuged and the pellets were lysed with RIPA buffer containing protease and phosphatase inhibitors. Tissue aliquots containing 500 µg of protein were incubated with the primary antibodies on a rotary apparatus for 1 h at 4°C. The immune complexes were washed three times in PBS buffer for 3 min each time. After the third wash, immunoprecipitates were re-suspended in SDS-PAGE sample buffer containing loading dye.

For Western blotting, 80 µg aliquots of protein were loaded onto 7.5% (w/v) or 10% (w/v) SDS-PAGE gels (Biorad, Hercules, CA), subjected to electrophoresis, and electrotransferred to nitrocellulose membranes. The membranes were blocked with 5% (w/v) non-fat milk powder in PBS for 30 min, followed by incubation with primary antibodies overnight at 4°C in PBS. Next, the blots were incubated with HRP-conjugated secondary antibodies at room temperature for 30 min**.** After three washes, for 10 min each time, with PBS containing 0.1% (v/v) Tween 20, membranes were incubated with the ECL reagent for 1 min at room temperature.

### 4. PTEN Plasmids and siRNA Transfection Experiments

HepG2 cells were plated in 12-well plates in DMEM supplemented with 10% (v/v) bovine serum. When the cells were 50–70% confluent, transfection was performed employing siRNAs targeting PTEN, PTEN or the PTEN mutant C124S. One day later, cells were serum-starved in DMEM for 12 hours and next stimulated with GH for different times as indicated. Cells were stimulated with insulin 10 minutes before lysis followed by harvested in RIPA buffer, centrifuged at 10,000 g at 4°C, and the supernatants were used for protein analysis using western blot.

### 5. Statistical Analysis

The significance of the data was determined by one-way analysis of variance (ANOVA) followed by Bonferroni test or Dunnett’s T test based on the Levene’s test of homogeneity of variances. P values less than 0.05 were considered as significant. All the statistical analyses were performed using SPSS version 17.0 (Chicago, IL, USA).

## Results

### 1. Chronic hGH Treatment Promoted Weight Gain and Induced Insulin Resistance

**Figure 1:**
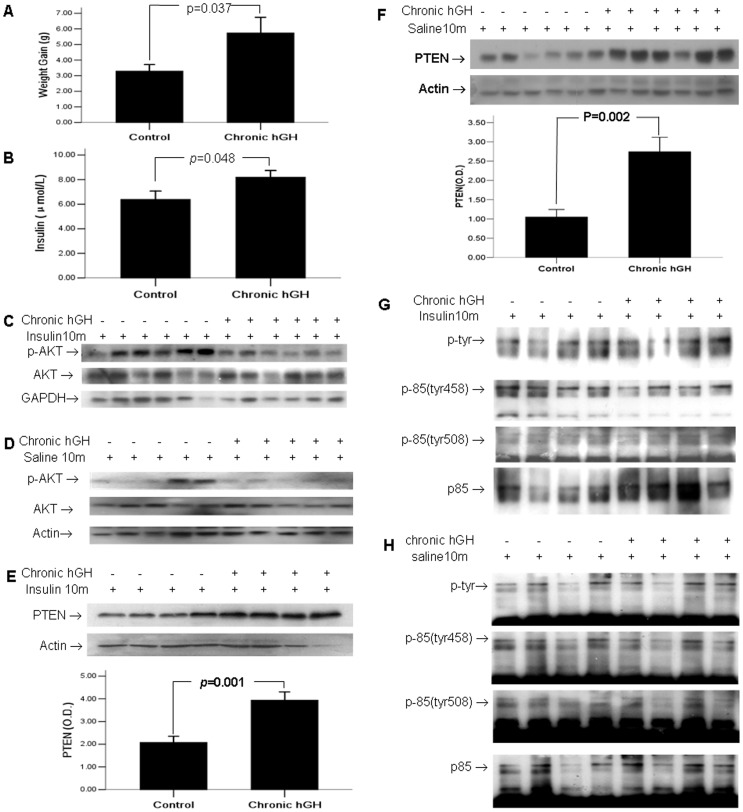
Chronic hGH treatment induced insulin resistance and enhanced the expression of PTEN. Mice were treated with daily hGH at 1 µg/g (controls received saline) for 3 weeks. (A) Fasting body weights were measured just before and after the completion of 3-week GH administration. (B) Plasma levels of Insulin were measured in mice without pre-sacrifice administration of insulin using ELISA Kit. (C) Hepatic Akt activity was examined in mice challenged by 10-minute insulin prior to sacrifice using Western blotting. (D) Hepatic Akt activity was examined in mice challenged by 10-min saline prior to sacrifice using Western blotting. (E) Hepatic PTEN expression was examined in mice challenged by 10-min insulin prior to sacrifice. (F) Hepatic PTEN expression was examined in mice challenged by 10-min saline prior to sacrifice. Liver lysates from mice challenged by 10-min insulin (G) or saline (H) prior to sacrifice were immunoprecipitated with antibody against the p85α subunit of PI3K and were blotted with antiserum specific, either for total tyrosine phosphorylation of p85, or for p85 phosphorylated at tyrosine 458 and tyrosine 508. The values in bar graphs were displayed as means ± standard errors.

As expected, compared with saline treated control group, 3-week daily administration of hGH caused approximately 2 folds greater degree of body weight gain (5.733±2.471 vs 3.286±1.11 g, P<0.05) (Fig. 1A). Insulin signaling was also impaired in the chronic hGH treated mice, with fasting serum insulin levels significant increased (8.187±1.89 vs 6.385±2.13 µIU/ml, p<0.05) (Fig. 1B). Consistently, when mice were challenged with exogenous insulin before sacrifice, the induced Akt activation was decreased in the chronic hGH treated group (p<0.05, the bar graph was not shown) (Fig. 1C). When mice were challenged with 10-min saline before sacrifice, no significant difference was observed in hepatic basal Akt activity between the chronic hGH treated group and the control group (p>0.05, the bar graph was not shown) (Fig. 1D).

### 2. Chronic hGH Treatment Enhanced the Expression of PTEN

It has been reported that deactivation of PTEN function promotes constitutive activation of insulin signaling, whereas enhanced PTEN function impairs insulin signaling [13], [16]–[20]. We found that PTEN was significantly increased in the chronic hGH-treated group, regardless of whether mice were challenged with 10-min insulin (Fig. 1E) or saline (Fig. 1F) before sacrifice (p<0.05). The phosphorylated state of p85 may functionally promote or inhibit the actions of PTEN [21], and plays a critical role in the activation of PI3K/Akt signaling. Phosphorylation of p85 subunit at tyrosine 458 and tyrosine 508 residues are found to be associated to the activation of PI 3 kinase [22]–[24]. However, we found no significant difference neither in total phospho-PI3K p85α nor in the phosphorylation of these two specific tyrosine residues, regardless of whether mice were challenged with 10-min insulin (Fig. 1G), or saline (Fig. 1H) before sacrifice (p>0.05).

### 3. The Effect of Chronic bGH Treatment on Healthy Mice

Human GH has been widely used in animals. However, hGH binds avidly to both GH and prolactin receptors in non-primates, so the experiments above using hGH have not differentiated the effects of GH versus the effects of PRL. Therefore, we further examined chronic effect of bovine GH in mice.

**Figure 2:**
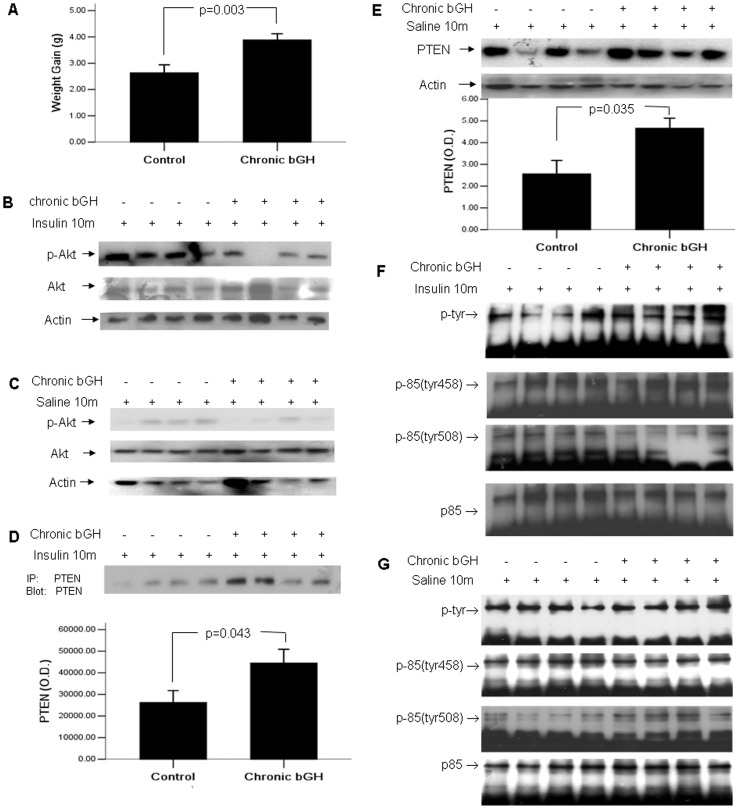
Chronic bGH treatment led to insulin resistance. Mice were treated with bGH at 1 µg/g (controls received saline) daily for 3 weeks. (A) Fasting body weights were measured just before and after the completion of 3-week GH administration. (B) Hepatic Akt activity was examined in mice challenged by insulin 10 minutes prior to sacrifice using Western blotting. (C) Hepatic Akt activity was examined in mice challenged by 10-min saline prior to sacrifice. (D) Liver lysates from mice challenged by 10-min insulin prior to sacrifice were immunoprecipitated with antibody against PTEN, and were blotted with the same antibody. (E) Hepatic PTEN expression was examined in mice challenged by 10-min saline prior to sacrifice. Liver lysates from mice challenged by 10-min insulin (F) or saline (G) prior to sacrifice were immunoprecipitated with antibody against the p85α subunit of PI3K and were blotted with antiserum specific, either for total tyrosine phosphorylation of p85, or for p85 phosphorylated at tyrosine 458 and tyrosine 508. The values in bar graphs were displayed as means ± standard errors.

Similar to our previous results, mice with chronic bGH treatment had a significant increase in weight gain (3.964±0.937 vs 2.582±1.29 g, p<0.01) (Fig. 2A) and expression of PTEN regardless of whether mice were challenged with 10-min insulin (Fig. 2D) or saline (Fig. 2E) before sacrifice (p<0.05). Akt activation was inhibited in chronic GH treated group when mice were challenged with 10-min insulin before sacrifice (p<0.05, the bar graph was not shown) (Fig. 2B). When mice were challenged with 10-min saline before sacrifice, basal Akt activation appeared to be slightly lower in the chronic GH treated group (p>0.05, the bar graph was not shown) (Fig. 2C). Non-significant change was found in total phosphorylation of p85 subunit, tyrosine 458 or 508, neither in mice treated with 10-min insulin (Fig. 2F) or saline (Fig. 2G) before sacrifice (p>0.05).

### 4. The Effect of Chronic hGH Treatment on STZ-induced Type I Diabetic Mice

GH and insulin are two important hormones in life and have a close relationship in the regulation of the cells signals. To test whether the increased expression of PTEN after chronic GH treatment was involved in the interaction of GH with insulin, we conducted our test in STZ-induced type I diabetic mice.

**Figure 3:**
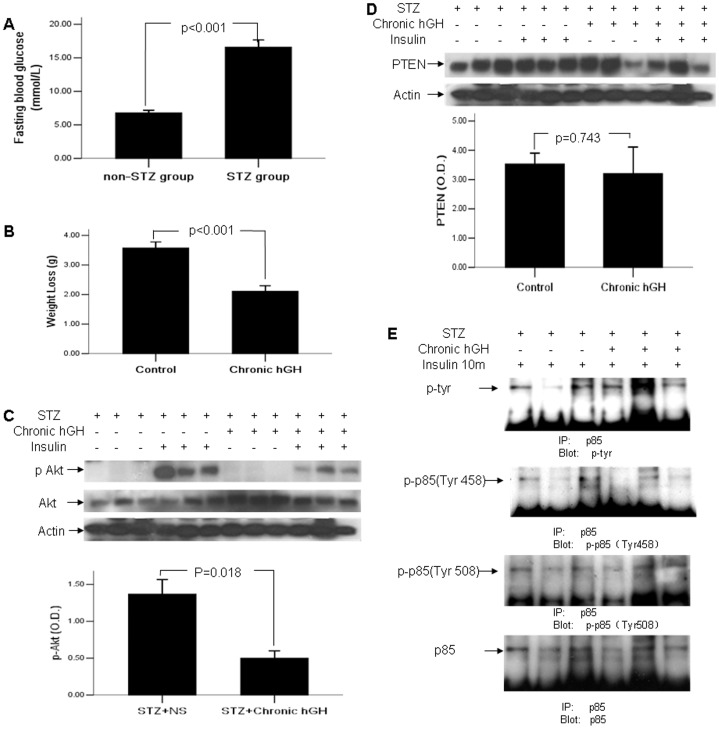
Chronic hGH induced insulin resistance without increased PTEN expression in STZ-induced diabetic C57BL/6 mice. (A) Fasting morning blood glucose of non-STZ and STZ group were measured by OneTouch® UltraVue™ glucometer prior to GH treatment. (B) Fasting body weights were measured before and after the completion of 3-week GH administration. (C) Hepatic Akt activity was examined in mice challenged by 10-minute insulin prior to sacrifice using Western blotting. (D) Hepatic PTEN expression was examined by Western blotting. (E) Liver lysates were immunoprecipitated with antibody against the p85α subunit of PI3K and were blotted with antiserum specific, either for total tyrosine phosphorylation of p85, or for p85 phosphorylated at tyrosine 458 and tyrosine 508. The values in bar graphs were displayed means ± standard errors.

As was shown in Fig. 3A, the fasting blood glucose was significantly increased after 3-day consecutive daily injection of STZ (16.59±4.40 vs 6.78±1.33 mmol/L, p<0.01), and a total of 17 mice with fasting blood glucose of over 11.1 mmol/L were selected and randomly divided into control and chronic hGH group.

Both groups of diabetic mice had weight loss, while chronic hGH administration caused less degree of weight loss (2.111±0.562 vs 3.575±0.575 g, p<0.05) (Fig. 3B). Akt activation in STZ-induced diabetic mice was significantly low (p<0.05) (Fig. 3C). Unexpectedly, the chronic GH treated mice had similar or even slightly lower level of PTEN compared to the control mice (p>0.05) (Fig. 3D). It indicated that the increased level of PTEN induced by chronic GH treatment in healthy mice might be via the interaction between PTEN and insulin. Similarly, as was shown in Fig. 3E, total phospho-tyrosine of PI3K p85 subunit displayed non-significant change in chronic GH group, neither in tyrosine 458 or tyrosine 508 residues (p>0.05).

### 5. The Effect of Chronic hGH Treatment on Mice with Hypoinsulinemia Induced by Prolonged Fasting

**Figure 4:**
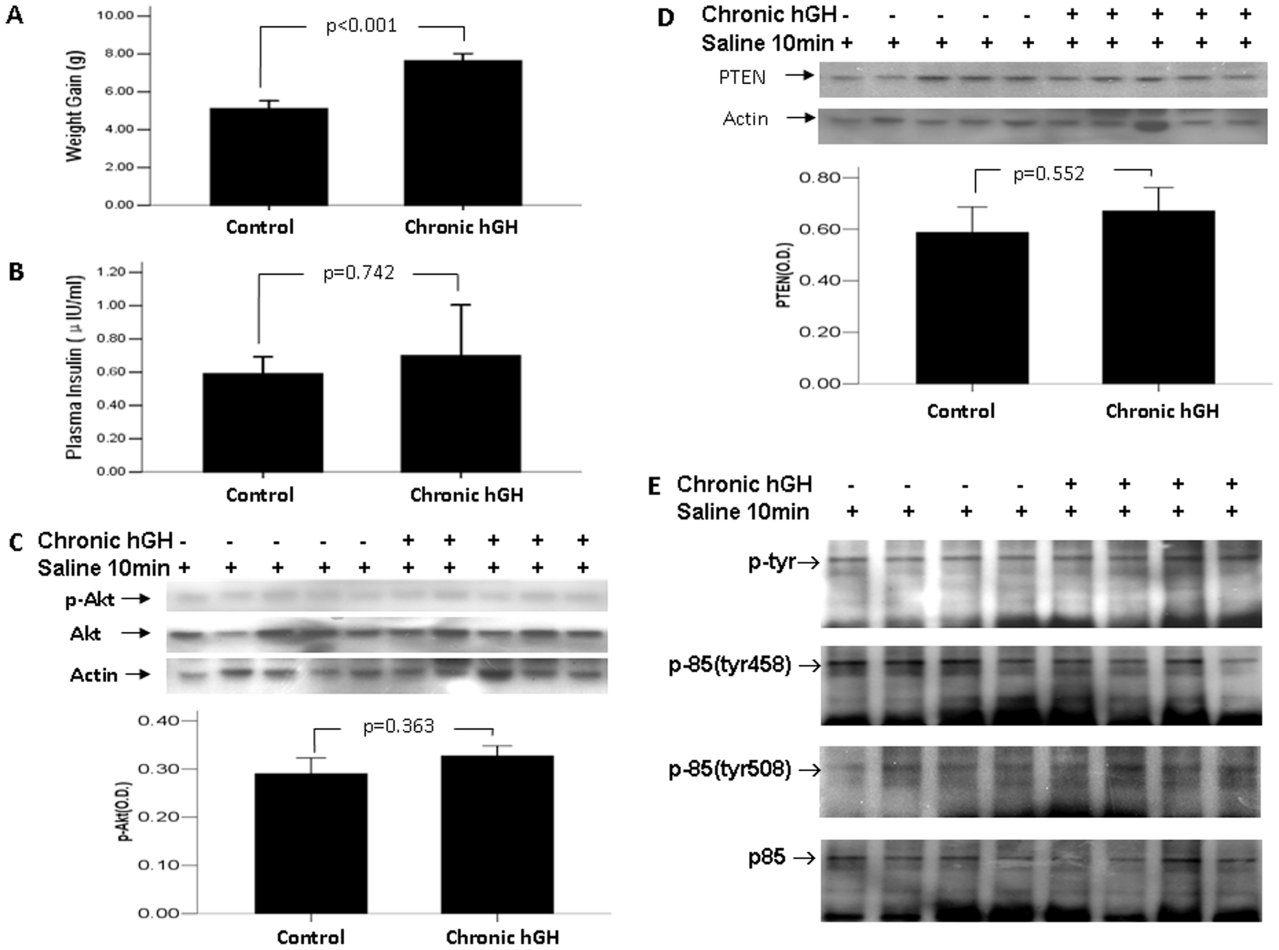
The effect of chronic hGH treatment on mice with hypoinsulinemia induced by prolonged fasting. Mice were treated with daily hGH at 1 µg/g or saline for 3 weeks, and subjected to fasting in the last 3 days. (A) Fasting body weights were measured just before and after the completion of 3-week GH administration. (B) Plasma insulin levels were measured using ELISA Kit. (C) Hepatic Akt activity was examined using Western blotting. (D) Hepatic PTEN expression was examined using Western blotting. (E) Liver lysates were immunoprecipitated with antibody against the p85α subunit of PI3K and were blotted with antiserum specific, either for total tyrosine phosphorylation of p85, or for p85 phosphorylated at tyrosine 458 and tyrosine 508. The values in bar graphs were displayed means ± standard errors.

Given that prolonged fasting can cause low insulinemia [25], we used this more physiological model to examine whether the interaction of GH with insulin was involved in the increased expression of PTEN after chronic GH treatment. In this experiment, mice received 3-week daily administration of saline or hGH (1 µg/g body weight) and in the last 3 days, were exposed to 3-day fasting. Mice with chronic bGH treatment had a significant increase in weight gain (7.636±1.264 vs 5.108±1.430 g, p<0.05, Fig. 4A). Both the chronic GH-treated and control mice had hypoinsulinemia without significant difference (0.591±0.249 vs 0.699±0.746 µIU/ml, p>0.05, Fig. 4B). The change in the Akt activity (Fig. 4C), PTEN expression (Fig. 4D), or the phosphorylation of p85 (Fig. 4E) was not significant between the chronic GH-treated mice and control mice (p>0.05).

### 6. Prolonged GH Exposure Inhibited Akt activity in HepG2 Cells

**Figure 5:**
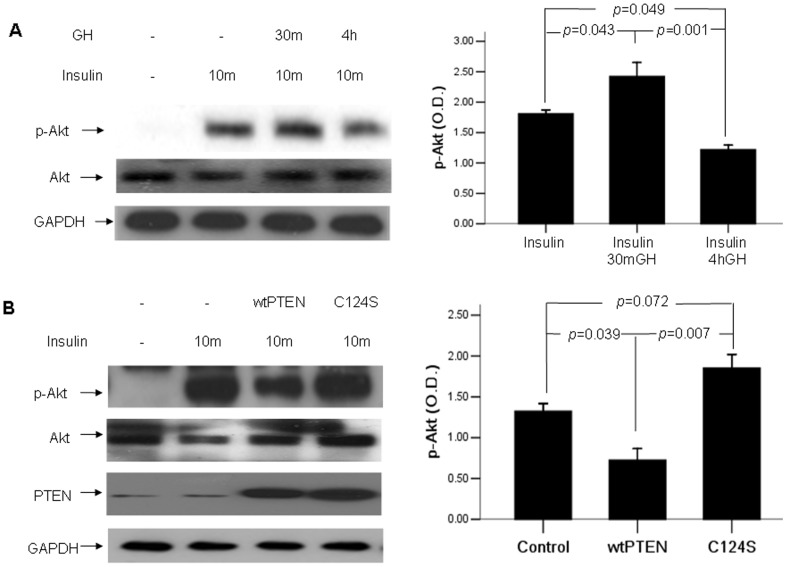
The effect of prolonged GH exposure and PTEN on Akt activity in HepG2 cells. (A) HepG2 cells were pretreated with GH or 30 min or 4 hours and harvested after insulin stimulation for 10 min. (B) Cells were transfected with wild type PTEN or PTEN mutant C124S, and harvested after insulin stimulation for 10 min. Densitometry absorbance values were averaged after they had been normalized to Akt for equal loading.

In order to further explore the effect of chronic GH on insulin signaling, we performed experiments *in vitro* using the HepG2 cell line to validate our *in vivo* findings. Fig. 5A showed that acute exposure to hGH significantly enhanced Akt phosphorylation (p<0.05), whereas prolonged exposure to GH significantly inhibited Akt phosphorylation (p<0.05) (Fig. 5A).

To examine the role of PTEN in insulin signaling, HepG2 cells were transfected with wtPTEN and its mutant C124S. Akt activity was significantly depressed in cells overexpressing wtPTEN (p<0.05), whereas it was slightly increased in cells overexpressing C124S (p>0.05) (Fig. 5B).

### 7. Knockdown of PTEN Gene Prevented Chronic GH-triggered Insulin Resistance in HepG2 Cells

**Figure 6:**
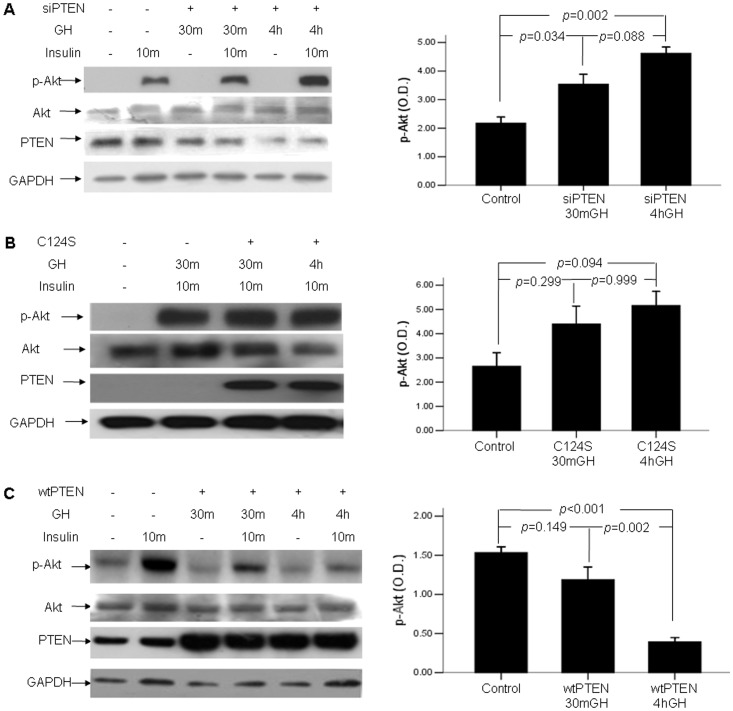
Knockdown of PTEN gene disrupted chronic GH induced insulin resistance. Cells were transfected with siRNA targeted for PTEN (A), PTEN mutant C124S (B) or wtPTEN (C), and were stimulated with GH and Insulin as stated in Methods and materials. Densitometry absorbance values were averaged after they had been normalized to Akt for equal loading.

We performed experiments to obtain direct evidence of the role of PTEN in the insulin resistance upon prolonged exposure to GH. Fig 6A showed that knockdown of PTEN gene with siRNA prevented the insulin resistance upon prolonged exposure to GH (p<0.05). Similarly, over-expressing C124S disrupted insulin resistance upon prolonged GH exposure (p<0.05) (Fig. 6B). To the contrary, cells overexpressing wtPTEN had similar even slightly greater insulin resistance upon prolonged GH exposure (p<0.05) (Fig. 6C).

## Discussion

There has been a long studied discrepancy between the effect of acute and chronic GH on insulin sensitivity, which remains an unsolved important mechanistic question [26]. We identified a novel mechanism to explain this discrepancy. We found that the PTEN was closely associated with the development of hepatic insulin resistance upon chronic GH treatment, at least in healthy mice.

A PTEN null mutation in mice results in embryonic lethality, precluding studies of disease mechanisms [27]. However, tissue-specific PTEN knockout models have shown that increased activity of PTEN prevented the development of insulin resistance. Muscle-specific deletion of PTEN prevented the development of insulin resistance and diabetes induced by high-fat diet [28]. Reduction of PTEN expression in liver and fat, achieved via systemic administration of a PTEN antisense oligonucleotide, protected db/db mice from developing diabetes [29]. To the contrary, PTEN expression was increased after chronic GH treatment in our study, and the animal accordingly presented insulin resistance. The association of the increased PTEN activity with insulin resistance after chronic GH treatment, may have implications in the understanding that PTEN polymorphisms have been identified in type 2 diabetes patients [14].

Growth hormone is an important modulator of insulin sensitivity. Various studies of GH excess, deficiency or resistance have demonstrated that liver is a major site of GH-induced insulin resistance [2], [30]–[32]. For example, excess of GH leads to reduced insulin receptor levels in liver, which may be associated with GH-induced hepatic insulin resistance [33]. However, these events of the GH/insulin paradigm could be more complex, and be modulated by various conditions. For example, in the obese cases, low-grade chronic inflammation, regulation of liver-spleen axis and insulin-like growth factor I, may implicate hepatic insulin resistance and metabolic syndrome [34], [35]. In present study, the pathological situation of type I diabetes may modulate GH induced insulin resistance, and result in a different mechanism from that in healthy mice.

Insulin and GH are closely related. So far, few studies exploring the role of PTEN in insulin resistance have been conducted without involving the effect of insulin. To explore this mechanism, we used STZ to inhibit the secretion of endogenous insulin. We showed that mice lacking endogenous insulin induced by STZ had similar insulin resistance after chronic GH treatment, but PTEN expression did not change. Moreover, in the physiological animal model, chronic GH-treated mice with hypoinsulinemia induced by prolonged fasting had only a marginal increase in PTEN expression compared to the control mice. These results indicated that the regulation of PTEN activity might be via the interaction of GH with insulin, and plays an important role in the chronic GH-induced insulin resistance. This may also indicate that hypoinsulinemia due to prolonged fasting might reduce the accumulation of PTEN induced by chronic GH. It may explain why chronic GH treatment did not increase PTEN expression in diabetic mice.
